# Review of the role of the nervous system in glucose homoeostasis and future perspectives towards the management of diabetes

**DOI:** 10.1186/s42234-018-0009-4

**Published:** 2018-07-04

**Authors:** Amparo Güemes, Pantelis Georgiou

**Affiliations:** Centre for Bio-Inspired Technology, Department of Electrical and Electronic Engineering, Imperial College London, South Kensington Campus, London, UK

**Keywords:** Diabetes management, Bioelectronic medicine, Neuromodulation, Insulin sensitivity, Pancreatic secretion

## Abstract

Diabetes is a disease caused by a breakdown in the glucose metabolic process resulting in abnormal blood glucose fluctuations. Traditionally, control has involved external insulin injection in response to elevated blood glucose to substitute the role of the beta cells in the pancreas which would otherwise perform this function in a healthy individual. The central nervous system (CNS), however, also plays a vital role in glucose homoeostasis through the control of pancreatic secretion and insulin sensitivity which could potentially be used as a pathway for enhancing glucose control. In this review, we present an overview of the brain regions, peripheral nerves and molecular mechanisms by which the CNS regulates glucose metabolism and the potential benefits of modulating them for diabetes management. Development of technologies to interface to the nervous system will soon become a reality through bioelectronic medicine and we present the emerging opportunities for the treatment of type 1 and type 2 diabetes.

## Background

Diabetes is a chronic metabolic disease which results in elevated blood glucose. It currently affects over 422 million people worldwide and is forecast to be the 7th leading cause of death in 2030 (World Health Organization 2016). Persistent elevated glucose levels (hyperglycemia) due to insufficient treatment is of increasing concern given its association with a number of secondary complications such as ketoacidosis, blindness, nephropathy and heart disease (Nathan 2014). Furthermore, current treatments have also been shown to increase the frequency of low blood glucose (hypoglycemia) (Cryer et al. 2003), therefore impacting the quality of life of the sufferers. As a result, significant efforts have been focused over the last decade on improving diabetes management through the introduction of technology to reduce hyper and hypoglycemia.

Diabetes is generally classed as type 1 or type 2 depending on the cause of the disease. Type 1 diabetes is an autoimmune disease that causes destruction of the pancreatic *β*-cells. This results in the absence of insulin secretion, the hormone responsible for glucose absorption, and therefore poor regulation of plasma glucose levels. Throughout the years, several techniques have been developed in order to improve the control of glucose for type 1 diabetes. The most widely adopted approach relies on external subcutaneous injection of insulin through the use of an insulin pen or infusion pump (Haidar et al. 2015). However, this method is still open-loop (i.e. it is not an automated system) that requires a lot of manual effort from the user and, as a result, most patients still have suboptimal glycemic control (Barnard et al. 2015). Alternatively, systems that respond to changes in blood glucose concentrations by automatically modulating insulin delivery have been developed. These are known as automated closed-loop insulin delivery systems, also described as an artificial pancreas (AP), and their use has resulted in improved glucose control and reduced risk of nocturnal hypoglycemia in comparison with previous techniques (Haidar et al. 2015). However, challenges such as the delay of insulin action during meals as a result of the subcutaneous infusion (Gingras et al. 2018) or the hypoglycemic events induced by exercising (De Bock et al. 2017), still have to be resolved in order to achieve the best glycemic control.

The second class of diabetes, type 2, is characterised by a chronic hyperglycemia resulting from defects in insulin action, which leads to a reduction of insulin sensitivity and insulin resistance. As a result several methods have been developed to try to modulate insulin sensitivity in these patients with the aim of reversing the disease. Among them, changes in nutrition and exercise (Weickert 2012) and drug therapies oriented to take control over the inflammatory processes that underlie the insulin resistance (Lamb and Goldstein 2008) are the most widely studied treatments. However, as reviewed by Gao and Ye (2012) the efficacy of anti-inflamatory treatments has not been proven (Gao and Ye 2012).

The challenges encountered in diabetes management are a consequence of the great complexity of the biological mechanisms for glucose control. In fact, many biological substances in addition to insulin, such as other hormones and neurotransmitters, interact with each other to ensure a robust and tight regulation of the glucose in the blood (Chandra and Liddle 2009). In particular, the brain, acting through the peripheral innervation to the different organs, has been found to have a crucial role in maintaining glucose homoeostasis. Evidence of this important glucose regulation mechanism dates back to the work of the physiologist Claude Bernard, who for the first time showed a causal relationship between brain stimulation of the fourth ventricle in the hindbrain and an increase in plasma glucose levels (Bernard 1865). It is therefore not surprising that recent research in the field of diabetes has been exploring the neural mechanisms that are involved in glucose control, initiating new perspectives for studying both type 1 and type 2 diabetes (Chandra and Liddle 2014; Rodriguez-Diaz and Caicedo 2014; Rosario et al. 2016).

Within this context, over the last few years, a novel emerging field called bioelectronic medicine has been introduced as an alternative treatment to using pharmaceuticals. Bioelectronic medicine uses electrical stimulation to modulate the neural activity of peripheral nerves, which in turn target specific organs and evoke a response towards treating a specific disorder or restoring lost biological functions (Birmingham et al. 2014). It has been successfully demonstrated in the treatment of traumatic brain injury, stroke and inflammatory disease (Koopman et al. 2014; Levine et al. 2014; Sundman and Olofsson 2014; Borovikova et al. 2000). Following this trend, we believe that advances in technologies for modulating the neural pathways that are involved in controlling glucose homoeostasis and the hormonal secretion by the pancreas may also soon become a reality and thus bioelectronic medicine could become a viable option for the treatment of diabetes.

Towards this objective, this comprehensive review shows that there is potential for improved glucose homeostasis using bioelectronic medicine by identifying the various areas in the central and peripheral nervous system which could impact glucose control. In the first part, we provide an overview of the central nervous system’s regions and signalling mechanisms that are involved in glucose homeostasis. We focus especially on their impact on insulin sensitivity (i.e. the effect of insulin in the balance of glucose production and uptake). The second part focuses on the control of hormonal secretion by the endocrine pancreas through the peripheral nervous pathways. Each section is followed by a discussion on the therapeutic opportunities of electrically modulating (either via stimulation or inhibition) these brain regions and peripheral pathways for diabetes management.

## Overview of the role of the CNS in control of glucose homeostasis

### Principal CNS regions and neuronal populations

**Fig. 1:**
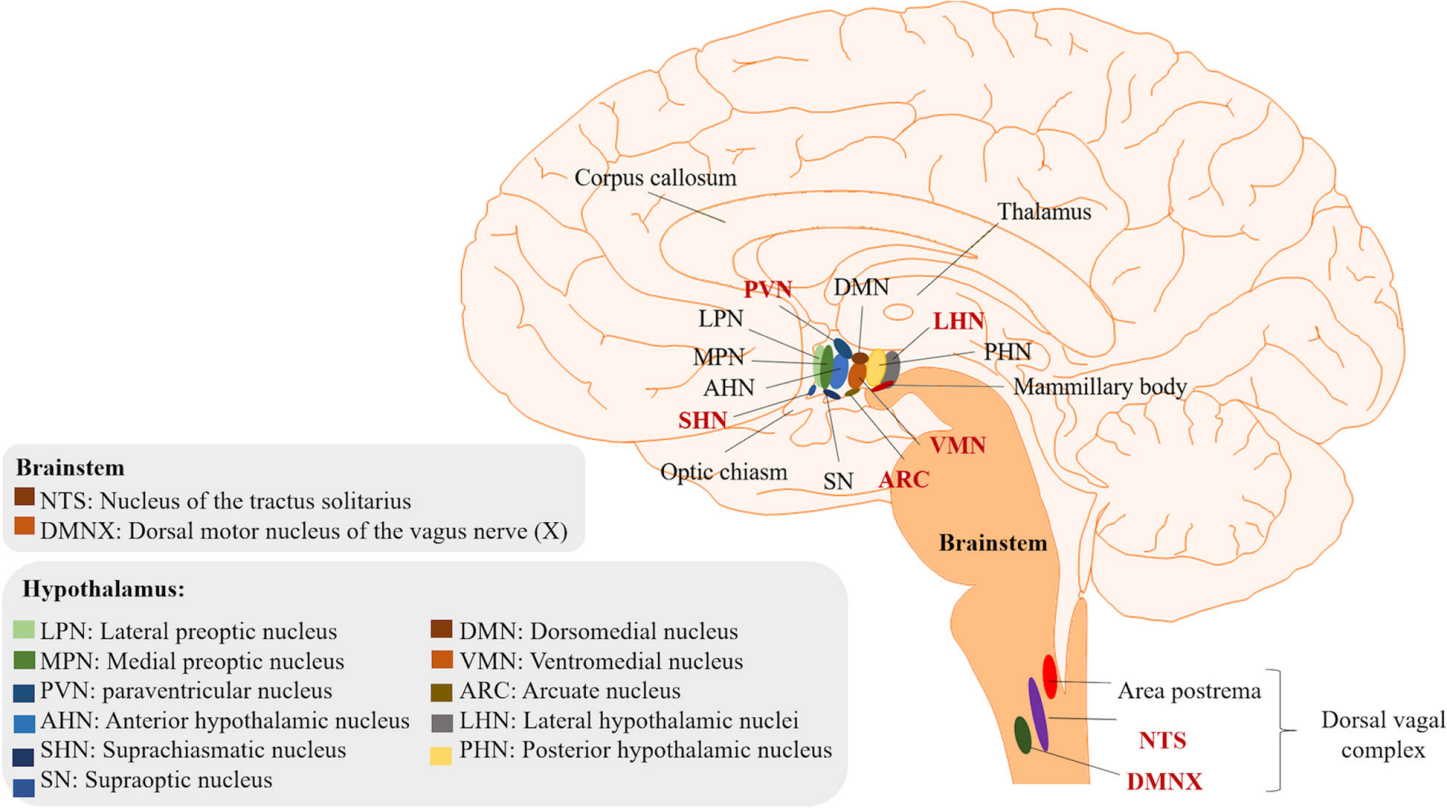
Key hypothalamic nuclei and other areas involved in glucose homeostasis. Representation of a sagittal section of a human brain, where the most relevant nuclei of the hypothalamus and the brain stem involved in control of glucose production and uptake are depicted

One of the brain areas that is most involved in the regulation of glucose homeostasis is the hypothalamus, located in the diencephalon (Zhang and van den Pol 2016). To have a better understanding of its physiology and role in glucose homeostasis, it is important to briefly present its anatomy. There are eleven major nuclei in the hypothalamus (see Fig. 1). Four of them have been found to have key roles in neuroendocrine regulation: i) the arcuate nucleus (ARC), located next to the third ventricle in the mediobasal hypothalamus, ii) the paraventricular nucleus (PVN), located in the periventricular zone, iii) the ventromedial nucleus of the hypothalamus (VMH), and iv) the lateral hypothalamic nucleus (LHN) (Broberger and Hökfelt 2001; Shin et al. 2017; Williams et al. 2001). The suprachiasmatic nucleus (SCN), involved in circadian timing, as well as the anterior and preoptic nuclei, involved in control of the autonomic nervous system, have also been proven to play a role in the control of energy homeostasis (Dougherty 2018). In addition to the hypothalamus, there are other brain regions, such as the brainstem, that contain nuclei which are also implicated in the regulation of the body’s energy homeostasis (Williams et al. 2001).

#### Arcuate nucleus of the hypothalamus

The ARC has important implications in the control of glucose homeostasis, including the regulation of food intake and energy balance (Wang et al. 2004; Elmquist and Marcus 2003). For example, some studies have demonstrated that damages in this area in humans result in hyperphagia and obesity (Broberger and Hökfelt 2001; Williams et al. 2001; Bray and Gallagher 1975).

The ARC has discrete neuronal populations which express neurotransmitters that mediate specific effects on food intake and energy expenditure and that are regulated by specific signals of nutritional state (Broberger and Hökfelt 2001; Williams et al. 2001; Wang et al. 2004; Yi et al. 2010). Among them, two populations of neurons stand out: the orexigenic neuropeptide Y/agouti-related peptide (NPY/AgRP) neurons and the anorexigenic pro-opiomelanocortin/cocaine-amphetamine related transcript (POMC/CART) neurons. Activation of AgRP and NPY neurons have been reported to promote anabolic processes that lead to a markedly increase of food intake (Broberger and Hökfelt 2001; Shin et al. 2017; Williams et al. 2001; Wang et al. 2004; Krashes et al. 2011; Billington and Levine 1992), whereas their ablation results in weight loss in mice (Gropp et al. 2005; Luquet et al. 2005). On the contrary, activation of POMC neurons favours catabolic processes, reducing appetite and food intake, whereas their inhibition increases feeding (Broberger and Hökfelt 2001; Shin et al. 2017; Wang et al. 2004; Koch et al. 2015). Recently, a new population of neurons in the ARC have been discovered, tyrosine hydroxylase (TH) neurons, which have orexigenic effects (Zhang and van den Pol 2016). Finally, the ARC also possesses glucosensing neurons (Wang et al. 2004; Spanswick et al. 1997) that take part in the neural circuitry of energy control.

It is important to note that the blood-brain barrier of the ARC is permeable to several hormones that are related to food intake and glucose homeostasis (Wang et al. 2004; Broadwell and Brightman 1976; Ganong 2000). For example, leptin and insulin receptors are widely expressed in ARC neurons (Broberger and Hökfelt 2001; Ahima et al. 2000; Cheung et al. 1997; Elmquist et al. 1998; Schwartz et al. 1997; Van Houten et al. 1980). As a result, the ARC is thought to be a primary receptive region for insulin and leptin and it acts a metabolic ’sensor’ that integrates endocrine information about the body energy homeostasis (Broberger and Hökfelt 2001; Qatanani and Lazar 2007).

##### NPY/AgRP neurons

The contribution of NPY/AgRP neurons to metabolic homeostasis has been widely studied and is well characterized. This population synthesises and secretes two neuropeptides, AgRP and NPY, that have potent orexigenic effects (Broberger and Hökfelt 2001; Billington and Levine 1992; Ollmann et al. 1997; Levin and Dunn-Meynell 1997; Shi et al. 2013; Stanley and Leibowitz 1984). For this reason, its activation powerfully promotes feeding and altering its activity deeply affects peripheral glucose homeostasis (Steculorum et al. 2016).

AgRP is a 132-amino acid protein whose overexpression results in obesity (Ollmann et al. 1997). In addition, recent studies have described that acute activation of AgRP neurons can cause insulin resistance through an impairment of the insulin-stimulated glucose uptake into brown adipose tissue (BAT). The mechanism that underlies this impairment is related to a reprogammation of the gene expression in BAT tissue towards a myogenic signature, by increasing the expression of myostatin (Mstn) (Steculorum et al. 2016). In fact, this loss of insulin sensitivity was restored interfering with the myostatin activity (Steculorum et al. 2016). Myostatin is a growth factor expressed in skeletal muscle and adipose tissue that negatively regulates skeletal muscle mass and whose expression has been linked to abnormal glucose metabolism (Guo et al. 2009). For instance, inhibition of myostatin signaling in skeletal muscle resulted in increased lean mass, decreased fat mass and improved glucose metabolism, insulin sensitivity (*S*_*I*_) and glucose uptake (Guo et al. 2009). These results reveal the importance of the neural control of the myostatin expression both in BAT and skeletal muscle for the regulation of insulin sensitivity.

The mechanisms by which the AgRP population controls feeding and insulin sensitivity are related to two different but overlapping projections, as recent optogenetic circuitry mapping techniques have revealed (Steculorum et al. 2016). Stimulation of projections to the lateral hypothalamus (*AgRP*→*LHN* projections) promotes feeding (Steculorum et al. 2016) while activation of the projections to the anterior bed nucleus of the stria terminalis (*AgRP*→*aBNST*6^*vl*^ projections) mediate the activation on BAT-Mstn expression, and therefore increases insulin resistance (Steculorum et al. 2016). However, activation of this projection did not report any change in feeding behaviour (Steculorum et al. 2016). It is also worth remarking that the control of AgRP neurons over insulin sensitivity and glucose metabolism has been found to be independent of the melanocorin pathway (i.e, there is no involvement of POMC neurons) (Steculorum et al. 2016; Wu et al. 2008).

NPY is a 36-amino acid neuropeptide whose expression is upregulated by fasting (Broberger and Hökfelt 2001; Levin and Dunn-Meynell 1997). The effects of activation of the NPY population on glucose metabolism include increased food intake and increased white fat storage (Broberger and Hökfelt 2001; Billington and Levine 1992; Shi et al. 2013; Stanley and Leibowitz 1984). Aditionally, NPY derived from the ARC is critical in the control of sympathetic outflow and BAT function (Shi et al. 2013). In fact, a recent study has reported that an increase of NPY from the ARC decreases the sympathetic outflow that controls the BAT thermogenesis via TH neurons (Yi et al. 2010). In more detail, NPY induced a deep reduction of tyrosine hydroxylase expression in the PVN (through projections from ARC to PVN) (Yi et al. 2010), in the locus coeruleus (LC) and in other regions in the brainstem. This led to a reduced sympathetical innervation to BAT and a decreased thermogenesis (Shi et al. 2013). NPY neurons have also been shown to increase hepatic glucose production (HGP), probably by reducing hepatic insulin sensitivity via increase of the sympathetic tone of the liver (Yi et al. 2010; Van Den Hoek et al. 2008). In addition, intracerebral infusion of NPY was able to reduce the inhibitory effects of insulin on hepatic glucose production (Van Den Hoek et al. 2008).

Apart from the widely studied effect of AgRP/NPY neurons in metabolic homeostasis, some research has been done in the last years to assess its implication in the temporal control of food intake. The SCN, which responds to light, is believed to be the primary circadian oscillator. However, the biological rhythms that arise in response to changes in food availability are known to persist even in the absence of the SCN (Tan et al. 2014; Stephan et al. 1979). This suggests that there is a food entrainable oscillator that is separate and independent of the light entrained oscillator (Stephan et al. 1979). Some work was carried out in the past to identify the anatomical sites of the food entrainable oscillator (Tan et al. 2014; Stephan et al. 1979). Results from a recent study suggest that AgRP neurons in the arcuate nucleus are a key site of this food entrained oscillator where peripheral and possibly other cues are detected independent of visual cues (i.e.light) and allow an animal to predict in time when nutrients become available to adjust their behavior accordingly (Tan et al. 2014).

##### POMC neurons

The central melanocortin system is well known for its role in regulating food intake and body weight (Elmquist and Marcus 2003). The *α*-Melanocyte-Stimulating Hormone (*α*-MSH) is the most important POMC-derived peptide involved in feeding and metabolism. It is a non-selective full agonist of all the melanocortin receptors except from the melanocortin receptor 2 (MC2R), which is exclusive for the adrenocorticotropic hormone (ACTH). It is remarkable that the two major populations of neurons in the ARC, AgRP/NPY and POMC, have antagonistic effects with regards to glucose homeostasis, clearly illustrated by the fact that AgRP acts as an endogenous antagonist of the melanocortin receptors 3 and 4 (MC3R/MC4R) (Yi et al. 2010; Ollmann et al. 1997). This antagonizing function is of great importance to adapt the hypothalamic-pituitary-thyroid (HPT) axis to the predominant energy status. As an example, in fasting conditions a reduction in *α*-MSH and an increase in AgRP is needed (Lechan and Fekete 2006).

In some studies, infusion of antagonists of MC3R and MC4R, which blocks the *α*-MSH pathway, resulted in no effects on glucose metabolism (Yi et al. 2010; Ruud et al. 2017). However, in other study, intracerebral infusion of *α*-MSH resulted in increased gluconeogenesis and hepatic glucose production (Yi et al. 2010; Lin et al. 2010). This excitatory mechanism could be inhibited by simultaneous infusion of antagonists of the MC3R/MC4R (Yi et al. 2010). In addition, it has been suggested that the hypothalamic MC3R/MC4R signaling pathway might mediate glucose production via mechanisms involving systemic leptin (Yi et al. 2010). Recent studies have also shown that activation of the melanocortin pathway results in increased sympathetic outflow enhancing white adipose tissue (WAT) lipolysis (Brito et al. 2007). This demonstrates the pivotal role of POMC neurons in the sympathetic regulation of adipose tissue (Shin et al. 2017; Scherer et al. 2012). Finally, the melanocortin system has also been found to have a role in regulating insulin release and insulin acion. For example, humans with MC4R mutations are extremely insulin resistant (Elmquist and Marcus 2003).

##### TH neurons

Arcuate TH neurons are involved in many neuronal circuits related to energy homeostasis. For example, studies of optogenetic stimulation of TH neurons of the ARC in mice showed an increase in food intake (Zhang and van den Pol 2016). In more detail, arcuate TH cells have projections to the PVN and when the former were stimulated, there was a co-release of the neurotransmitters dopamine and GABA to the neurons in the PVN. In the latter, dopamine excited orexigenic neurons that synthesize AgRP and NPY, but inhibited anorexigenic neurons that synthesize POMC (i.e. powerfully stimulating feeding) (Zhang and van den Pol 2016). Moreover, food deprivation was found to cause an increased firing frequency in arcuate TH neurons. This supports its important role in energy homeostasis, although it is not fully understood yet (Zhang and van den Pol 2016).

#### Ventromedial nucleus of the hypothalamus

The VMH is a key brain region involved in glucose regulation and energy homeostasis in mammals (Chowdhury et al. 2017; Coutinho et al. 2017; Routh 2010; Borg et al. 1997; Borg et al. 1994; Shimazu et al. 1966; Iigaya et al. 2017). In particular, the VMH has a crucial role in detecting hypoglycemic events and initiating the physiological counterregulatory responses to overcome it (Borg et al. 1997; Shimazu et al. 1966; Shimazu et al. 1991; Biggers et al. 1989; Chan et al. 2011). It involves the detection of glucose in this brain area, which then triggers specific mechanisms that end up with the release of glucagon and epinephrine (Chan et al. 2011). Studies of electrical stimulation of the VMH support this idea. Their results showed a marked increase in the circulating level of glucagon, together with a rapid rise in glucose levels (Shimazu and Ishikawa 1981; Stanley et al. 2016). On the contrary, the level of insulin remained unchaged during VMH stimulation, although it increased after cessation of stimulation (Shimazu and Ishikawa 1981).

The VMH contains a heterogeneous population of neurons. A subset of VMH neurons expresses the leptin receptor (Elmquist et al. 1998), and most of these neurons also express the steroidogenic factor-1 (SF1) receptor (Dhillon et al. 2006; Bingham et al. 2008). SF1 neurons in the VMH are required for maintenance of normal glucose and energy metabolism (Coutinho et al. 2017) and also mediate the anorexic and metabolic effects of leptin (Coutinho et al. 2017; Dhillon et al. 2006; Bingham et al. 2008). Leptin is a hormone predominantly made in adipose cells that helps to regulate energy balance by i) inhibiting hunger, ii) stimulating glucose uptake in some peripheral tissues, including red-type skeletal muscle and iii) enhancing endogenous glucose production by inducing glycogen phosphorylase activity in the liver. Thereby, it affects insulin sensitivity and helps to maintain proper blood glucose levels (Minokoshi et al. 1999; Toda et al. 2013). Exactly the same results were observed with the activation of SF1 neurons using DREADD (designer receptors exclusively activated by designer drugs) technology, which allows spatial and temporal control of the activity of specific neurons (Coutinho et al. 2017). Moreover, activation of SF1 neurons also reduced food intake and increased energy expenditure (Coutinho et al. 2017). Altogether, these results confirm the close relationship between leptin and SF1 neurons and supports the observation that leptin enhances the activity of this population of neurons (Coutinho et al. 2017; Dhillon et al. 2006).

Two major neurotransmitters are involved in the neural circuitry of the VMH, the inhibitory neurotransmitter GABA and the excitatory one glutamate. To begin with, VMH GABAergic neurotransmission has been extensively studied with respect to its role in glucose sensing. It is known that glucose deprivation alters GABA levels within the brain (Chan et al. 2007; Beverly et al. 2001). However, the evidence for its role in regulating glucose counterregulation remains somehow controversial. Some studies have reported that GABA acts within the VMH to modulate the magnitude of both the glucagon and epinephrine responses to hypoglycemia in nondiabetic rats (Chan et al. 2006). Accordingly, an increased GABAergic tone in the VMH has been observed to be an important contributor to counterregulatory failure in nondiabetic rats exposed to recurrent hypoglycemia (RH) (Chan et al. 2008). Moreover, in diabetic patients alterations in the capacity of the VMH to sense glucose deficit were shown to significantly contribute to the loss of glucagon secretion (Chan et al. 2011). GABA is greatly involved in the processes that lead to this impairment (Chan et al. 2011). In more detail, diabetes induced an increment in local GABA concentrations in the VMH, which in turn increased its GABAergic tone (Chan et al. 2011). This led to an impaired glucagon and epinephrine release during acute hypoglycemia. It has also been demonstrated that this counterregulatory impairment is reversed by specifically reducing excessive GABAergic inhibitory tone in the VMH (Chan et al. 2011). In conclusion, although the specific mechanisms responsible for these changes in the synthesis and release of GABA in the VMH remain to be established, this neurotransmitter has been suggested to have great implications in the defective glucose counterregulation in both the healthy and diabetic conditions (Chan et al. 2011).

Regarding the role of the glutamatergic neurotransmission in the VMH in glucose sensing (Chan et al. 2011; Chan et al. 2006), glutamate serves multiple purposes including the regulation of feeding and the modulation of the sympathetic nerve activity (SNA) (Iigaya et al. 2017; Lindberg et al. 2013; Abizaid and Horvath 2008; Guyenet et al. 2013; Narita et al. 1994). For instance, electrical stimulation of the VMH increased the sympathetic tone, while lesions in the VMH reduced it (Sakaguchi et al. 1988). In fact, the VMH affects the peripheral organs function via sympathetic innervation to the tissues (Yi et al. 2010; Shimazu et al. 1966; Gellman et al. 1981). Regarding its action in the liver, electrical stimulation of the VMH was found many years ago to increase hepatic glucose production (Yi et al. 2010; Shimazu et al. 1966; Gellman et al. 1981). This occurs by activation of glycogen phosphorylase (a key glycogenolytic enzyme) and a marked suppression of hepatic pyruvate kinase (PK) activity (a key glycolytic enzyme), resulting in hyperglycemia and a marked reduction in liver glycogen content (Yi et al. 2010; Shimazu et al. 1966; Shimazu et al. 1991). This result is consistent to those observed after electrical stimulation of the peripheral sympathetic splanchnic-nerve innervating the liver, supporting the idea of a VMH-splanich pathway for hepatic control (Shimazu et al. 1991).

Aditionally, the VMH activation increases glucose utilization and insulin sensitivity in some peripheral tissues. Electrical stimulation of the VMH has demonstrated an increase in glucose utilization in the interscapular BAT, heart, and skeletal muscle, but has not significantly affected the WAT, the brain and the diaphragm (Coutinho et al. 2017). The increased rate of glucose uptake in the BAT after VMH stimulation was suppressed almost completely by local sympathetic denervation, indicating again that the effect of VMH stimulation is also mediated by sympathetic nerves to the tissue (Shimazu et al. 1991). The fact that VMH stimulation does not increase insulin secretion from the pancreas (Shimazu and Ishikawa 1981), indicates that the effect of VMH stimulation on tissue glucose uptake is not mediated by insulin. On the contrary, it is probably mediated by sympathetic nerve activities, and therefore an insulin-independent mechanism of glucose transport. This may operate simultaneously in different insulin-sensitive tissues such as the skeletal muscle and BAT (Shimazu et al. 1991).

Moreover, it is known that SNA has a low-frequency modulation (typically ≤ 0.1 Hz) in addition to the frequency modulation which is related to the respiratory rhythm (Sakata et al. 2002; Baekey et al. 2008). Recent experiments have found a group of strong spontaneous low frequency burst-generating neurons (≤0.1 Hz) in the dorsolateral region of the VMH that might be involved in this low-frequency modulation of the SNA (Iigaya et al. 2017). However, the detailed neuronal mechanisms have not yet been determined (Iigaya et al. 2017).

Finally, a recent study has reported that VMH glutamate is also an integral feature of the counterregulatory response to hypoglycemia (Chowdhury et al. 2017). It is thought that RH impairs VMH glutamatergic neurotransmission by reducing glutamatergic neuronal metabolism and the concentration of interstitial glutamate in the VMH, which in turn, contributes to counterregulatory failure (Chowdhury et al. 2017). However, the underlying molecular mechanisms are not completely understood (Chowdhury et al. 2017).

#### Lateral hypothalamic nucleus

The LHN contains the primary orexinergic nucleus within the hypothalamus and widely projects to the entirety of the remainder of the hypothalamus, in particular to the posterior hypothalamus, the arcuate nucleus and the paraventricular hypothalamic nucleus (Malenka et al. 2009). Many studies of stimulation of the LHN have showed an action of this area over hepatic glucose metabolism (Yi et al. 2010; Shimazu et al. 1991). Early research in this field dating back 40 years reported a decrease in phosphoenolpyruvate carboxykinase (PEPCK) activity (involved in gluconeogenesis) after LHN stimulation, whereas the activity of the PK (related to the metabolism of glycogen) remained unaltered (Shimazu and Ogasawara 1975). On the contrary, most recent studies have proved its implications on glycogen metabolism in the liver, where VMH and LHN carry out reciprocal effects (Yi et al. 2010; Shimazu et al. 1991). In fact, stimulation of the LHN, contrary to VMH activation, resulted in increased hepatic glycogenesis by activation of the key enzyme glycogen synthetase (Yi et al. 2010; Shimazu et al. 1991). This result is consistent to those observed after electrical stimulation of the peripheral parasympathetic vagus-nerve innervating the liver (Shimazu et al. 1991), suggesting a LHN-parasympathetic control of the hepatic glycogenesis.

Moreover, evidences of the interaction between the LHN region and the pancreas via the pancreatic parasympathetic branch are broadly found in different experiments in the literature (Oomura and Kita 1981; Niijima 1986). Stimulation of different areas within the LHN had different effects on the pancreatic vagus nerve activity (Oomura and Kita 1981). For example, stimulation of the ventral LHN enhanced pancreatic vagus activity (Oomura and Kita 1981; Niijima 1986). Same conclusions were obtained by bilateral lesion of this area, which resulted in a decreased vagal activity (Niijima 1986). Moreover, the relationship between the activation of LHN and the pancreatic secretion is greatly dependent on the stimulating method. Electrical stimulation did not alter the plasma level of either insulin or glucagon (Shimazu and Ishikawa 1981). On the other hand, chemical stimulation with epinephrine induced a rise in the insulin level without any changes in the levels of glucagon and glucose (Shimazu and Ishikawa 1981).

Finally, up to date electrical stimulation of the LHN have not exhibited any significant direct effect on glucose uptake from either adipose or skeletal tissue (Shimazu et al. 1991; Shimazu and Ishikawa 1981). However, activation of the MC4R expressed in the LHN has been found to cause an increase of glucose uptake specifically into BAT, suggesting that *MC*4*R*^*LHN*^ signalling enhaces BAT glucose utilization via sympathetic pathways (Morgan et al. 2015).

#### Suprachiasmatic nucleus and paraventricular nucleus

The SCN is a small region of the hypothalamus, situated directly above the optic chiasm. As previously mentioned, it is well known for regulating many different body functions in a 24-h cycle (circadian rhythms). It is therefore not surprising that it plays a crucial role in the generation of the 24-h rhythm of plasma glucose (Cailotto et al. 2005). Activation of the sympathetic pathway have resulted in an increase of plasma glucose concentrations (Cailotto et al. 2005; Kalsbeek et al. 2004). In addition, studies of electrical stimulation of the SCN have also induced hyperglycemia, which was prevented by the use of sympathetic blockers or denervation (Yi et al. 2010; Kalsbeek et al. 2004; Fujii et al. 1989). This suggests a sympathetic mediation of the effects due to SCN activation. These hyperglycemic events were also reported in experiments removing the inhibitory input from the SCN to the PVN, suggesting an implication of the latter in generating the 24-h oscillations of plasma glucose (Lang 1995). This hyperglycemia comes as a result of i) direct (increased hepatic glucose production) (Cailotto et al. 2005; Kalsbeek et al. 2004) and indirect (through pancreatic secretion) (Nagai et al. 1988) stimulatory effects on glycogenolysis, ii) an increase in the activity of the hepatic enzyme glycogen phosphorylase alpha, which is involved in the glycogenolysis, and iii) a significant decrease in the liver glycogen content (Cailotto et al. 2005; Nagai et al. 1988).

#### Extrahypothalamic regions: nucleus of the tractus solitarius

The integration of information related to the body’s energy homeostasis does not occur solely in the hypothalamus. For example, meal-related satiety information is conveyed to the nucleus of the tractus solitarius (NTS) in the medulla, where vagally transmitted signals from the gastrointestinal tract converge (Williams et al. 2001). Some NTS neurones are glucose sensitive, while others express POMC, leptin receptors or the MC4R, giving evidence to its important function as a integration center of metabolic signals. The NTS also sends a dense projection to the LHN, reinforcening its contribution in the control of body energy (Williams et al. 2001).

### Metabolic and hormonal signalling in the brain

#### Glucose signalling

The ability of sensing glucose is of great importance for maintaining an optimal glucose homeostasis. Therefore, it is not surprising that the brain, which is the most important centre of control of our body, uses glucose sensing information as a major signal for integrating the neural activity and regulating the whole body metabolism (Levin et al. 1999; Thorens 2011). Glucose sensing cells can be found in the mouth, the hepatoportal vein area, the brainstem and the hypothalamus and are linked to each other by nervous connections. These neurons can be either glucose excited (GE) or glucose inhibited (GI) neurons that increase and decrease, respectively, their action potential frequency (APF) as extracellular glucose levels increase throughout the physiological range (Wang et al. 2004). They are thought to control activation or inhibition of either the parasympathetic and sympathetic nervous system that innervate pancreatic *α* and *β*-cells, regulating their hormonal secretion and cell number (Thorens 2011).

The mechanisms of glucose sensing by these neurons are still incompletely defined. Regarding the sensing mechanisms of the GE neurons, the glucose transporter GLUT2, which is requiered in pancreatic *β*-cells for glucose uptake, has also been found to be greatly expressed in hypothalamic and brainstem nuclei (Wang et al. 2004; Thorens 2011; Guillod-Maximin et al. 2004). For this reason they are thought to use similar mechanisms of extracellular glucose detection to that in pancreatic *β*-cells (Dunn-Meynell et al. 2002). Specifically, they use the GLUT2/glucokinase/KATP channel signalling pathway to control their firing activity (Thorens 2011; Dunn-Meynell et al. 2002). In addition, knockdown of GLUT2 transporter in the hypothalamic area has shown to decrease feeding and body weight gain, suppress the insulin response triggered by intracarotid glucose injection (Leloup et al. 1998) and reduce the glucagon secretion in response to insulin-induced hypoglycemia (Marty et al. 2005). On the other hand, the sensing system of GI neurons is not well established, though some studies found the activation of GI neurons in the VMH by low glucose to be mediated by AMPK-dependent phosphorylation (Thorens 2011). Depending on their location, the glucose sensing neurons have different functions on the glucoregulatory process:

##### Hypothalamic ARC nuclei.

Focusing solely on the arcuate nucleus, GI neurons are found predominantly in the medial ARC, whereas GE neurons are mainly located in the lateral ARC and are found to be intermixed with POMC neurons (Wang et al. 2004). Some studies have demonstrated that the latter change their firing rate in a dose-dependent fashion to physiological changes in extracellular glucose (Wang et al. 2004; De Vries et al. 2003). Under physiological conditions, brain glucose levels are generally aproximately 20–30% of the plasma levels (De Vries et al. 2003; Silver and Erecinska 1998). Thus, except in extreme hyperglycemic diabetic conditions, it is unlikely that extracellular brain glucose levels ever exceed 5 mmol/l. However, as previously mentioned, the blood-brain barrier has a higher permeability in the ARC (Wang et al. 2004; Broadwell and Brightman 1976; Ganong 2000). Thus, ARC glucose-excited neurons can be exposed to higher glucose levels than the rest of the brain. In fact, some studies have shown that glucosensing neurons in this region respond to increased glucose levels over 5 mmol/l (Wang et al. 2004).

It is important to highlight the modulating effect of other hormones and neurotranmitter over the activity of ARC GE neurons. To begin with, insulin has been found to modify their firing pattern in a glucose-dependent fashion. This excitatory effect of insulin might be due to an increase of the glucose transport into glucose-excited neurons (Wang et al. 2004; Kang et al. 2004). NPY and *α*-MSH also regulate the activity of ARC glucose-excited neurons in a fashion that is consistent to the roles of these peptides in the regulation of food intake, energy balance (Zhang and van den Pol 2016; Broberger and Hökfelt 2001; Wang et al. 2004; Elmquist 2001; Mounien et al. 2010): NPY inhibits and *α*-MSH stimulates ARC glucose-excited neurons (Wang et al. 2004; Cowley et al. 1999). On the other hand, leptin has no effect on ARC glucose-excited neurons (Wang et al. 2004). In conclusion, ARC glucose-excited neurons have a pivotal position as integrators of central and peripheral information of energy homoeostasis.

##### Hypothalamic VMH nuclei.

As previously stated, glucosensing neurons in the VMH are key elements in the counterregulatory response. Apart from the inherently glucose sensing neurons (Wang et al. 2004; Iigaya et al. 2017) (both GE and GI neurons), other neurons in the VMH were found to be able to alter their firing rate in response to a variety of extracellular glucose concentrations (Wang et al. 2004). However, the changes in their activity were due to presynaptic inputs from other glucosensing neurons whose cell bodies may reside outside the VMH, as opposed to directly sensing glucose levels. One possible location of these presynaptic glucosensing inputs to the VMH has been reported to be the ARC (Wang et al. 2004).

##### Brainstem nuclei.

In addition to those in the VMH, other glucosensing neurons in the brainstem have demonstrated to carry out an important role in the counterregulatory response to hypoglycemia. In fact, the brainstem might be the most physiologically relevant site of hypoglycemia detection and counterregulation (Thorens 2011).

To conclude, some studies suggest that impairment of these glucose-sensing mechanisms might be the origin of the metabolic deregulations that lead to obesity and type 2 diabetes, such as overeating, reduced energy expenditure, impaired suppression of glucagon secretion and reduced first phase insulin secretion (Thorens 2011).

#### Central insulin signalling

Both insulin and leptin have an important signalling role in central nervous mechanisms of glucose homeostasis and energy balance, illustrated by the great amount of their receptors that can be found in specific areas of the brain (Cheung et al. 1997; Elmquist et al. 1998; Schwartz et al. 1997; Van Houten et al. 1980), as in the ARC (Shin et al. 2017; Yi et al. 2010; Unger et al. 1989). Brain insulin exerts a different action depending on the target ARC population. As an example, insulin action on NPY/AgRP neurons results in their hyperpolarization (inhibition) causing a decreased NPY/AgRP gene transcription. On the contrary, its action on the POMC/CART neurons results in their depolarization (activation) leading to an increased POMC/CART transcription (Qatanani and Lazar 2007). It is also surprising that the action of insulin in the brain does not alter peripheral insulin signaling, but influences insulin action in peripheral tissues (Scherer et al. 2011; Ramnanan et al. 2011; Seeley and Tschöp 2006). Thus, hypothalamic insulin uses signaling pathways other than peripheral insulin in target organs such as WAT and liver to regulate insulin action (Scherer et al. 2012). As an illustration, insulin suppresses hepatic glucose production and lipolysis in adipose tissue through signaling in the mediobasal hypothalamus that alters parasympathetic and sympathetic outflow to these tissues, respectively (Shin et al. 2017; Scherer et al. 2011; Pocai et al. 2005).

Central insulin action in AgRP neurons has been found to be highly involved in regulating hepatic insulin action but without affecting peripheral glucose utilization (Shin et al. 2017). This result is in agreement with the observation that insulin infusion into the medial basal hypothalamus hyperpolarizes this region through activation of *K*_*ATP*_ channels resulting in hepatic glucose production inhibition (Lin et al. 2010), via the hepatic vagal innervation (Yi et al. 2010), but it does not increase glucose uptake in rats (Scherer et al. 2011). Moreover, recent studies in this area have demonstrated that mice lacking insulin receptors in AgRP neurons (AgRP IR KO mice) exhibited a mild hepatic insulin resistance (Shin et al. 2017; Qatanani and Lazar 2007; Lin et al. 2010; Könner et al. 2007), defined as a reduced ability to suppress hepatic glucose production, while the ability of insulin to suppress adipose tissue lipolysis remained intact (Shin et al. 2017). However, despite the hepatic insulin resistance, the glucose infusion rate (GIR) required to maintain euglycemia during the hyperinsulinemic clamps was not altered in AgRP IR KO mice, and importantly, they were also able to maintain normal glucose tolerance (Könner et al. 2007). This result suggest that the impairment of hepatic insulin action was moderate. Focusing in more detail on the mechanisms by which brain insulin action controls hepatic glucose production, it has been suggested that it occurs via induction of IL-6, which causes phosphorylation and activation of the transcription factor STAT-3 in the liver, which then regulates the expression of gluconeogenic genes (Lin et al. 2010; Inoue et al. 2006).

Although the previously mentioned studies have not reported any change in the glucose uptake by peripheral tissue, the role of insulin signalling for this process in AgRP neurons is not well defined (Shin et al. 2017). In fact, as Steculorum et al. have recently reported, the effect of hypothalamic insulin on glucose uptake varies depending on the type of tissue (Steculorum et al. 2016). Their results showed that insulin’s ability to promote glucose uptake into BAT was significantly reduced by 50% after AgRP neuron activation, while it remained unaltered in WAT and skeletal muscle (Steculorum et al. 2016).

Additionally, studies of the insulin signaling in POMC neurons have not provided evidence of significant alterations of neither hepatic glucose production nor glucose tolerance (Shin et al. 2017; Könner et al. 2007; Hill et al. 2010). In fact, intracerebral co-administration of melanocortin agonist failed to block the decrease in glucose production induced by central insulin (Gutiérrez-Juárez et al. 2004). However, it has been reported that mice lacking the insulin receptor in POMC neurons exhibit impaired adipose tissue insulin action, failing to suppress adipose tissue lipolysis (Shin et al. 2017). The latter is in agreement with studies that report that in healthy conditions, hypothalamic signaling acts by reducing sympathetic nervous system outflow to WAT, keeping a tight control of lipolysis and fatty acid (FA) release (Scherer et al. 2012; Scherer et al. 2011). In summary, these studies suggest the possibility that brain insulin indirectly controls hepatic glucose production by modifying the flux of glycerol and non-essential fatty acids (NEFAs) to the liver via suppression of lipolysis in WAT (Scherer et al. 2011). Moreover, in diabetic or obese patients, insulin failed to inhibit lipolysis from WAT and fatty acid levels were increased (Scherer et al. 2011). Uncontrolled FA release from WAT promotes lipotoxicity, which is characterized by inflammation and insulin resistance that can lead and worsen type 2 diabetes and can further impair brain insulin signaling, starting a vicious cycle (Scherer et al. 2012; Scherer et al. 2011).

It has also been revealed that insulin signaling in ARC influences hepatic insulin sensitivity via parasympathetic vagal nerves (Yi et al. 2010). Finally, a recent study has reported the impact of hypothalamic insulin signaling in lowering branched-chain amino acids (BCAA) levels. It was shown to activate the hepatic branched-chain alpha-keto acid dehydrogenase (BCKDH) complex, an enzyme catalyst for the second step of the BCAA catabolic pathway, which in turn lowered the plasma BCAA levels (Shin et al. 2014). BCAA levels, which are elevated in obesity/diabetes, are considered a sensitive predictor for type 2 diabetes because it is insulin resistance, and not hyperglycemia, the major cause of the increase in circulating BCAAs present in obesity and diabetes (Shin et al. 2014).

#### Central leptin signalling

Leptin action in the brain has been proven to be involved in hepatic glucose control by redistribution of intrahepatic glucose fluxes, increasing gluconeogenesis and surpressing glycogenolysis. However, it does not change total glucose production by the liver (Liu et al. 1998; Rossetti et al. 1997) in contrast to the central control of insulin that only acts to inhibit glucose production (Shin et al. 2017; Scherer et al. 2011). Previously, the close correlation between the effects of leptin and SF1-neurons in the VMH and how central leptin promotes the activity of this population of neurons were described (Coutinho et al. 2017; Dhillon et al. 2006). Central leptin has been also found to be involved in brain circuits that are closely related to the melanocortin pathway (Yi et al. 2010). Therefore, it is not surprising that although systemic leptin alone has not shown to alter hepatic insulin sensitivity, when it is centrally co-infused with muscarinic antagonists it can enhance hepatic insulin sensitivity (Gutiérrez-Juárez et al. 2004). Moreover, the effects of hypothalamic leptin signaling on hepatic insulin sensitivity could be blocked by selective hepatic vagotomy (Yi et al. 2010). In addition, the expression of leptin receptors in the ARC improved peripheral insulin sensitivity by promoting the suppression of glucose production (German et al. 2009). This result reinforces the idea that ARC projections to pre-autonomic neurons in the PVN are important for the transmission of the effect of both leptin and insulin on glucose production (Yi et al. 2010). Finally, it was shown that leptin deficiency induced severe insulin resistance that could be corrected with its infusion (Elmquist and Marcus 2003).

#### FAs signalling

In addition to leptin and insulin, there are other metabolic substances such as fatty acids that are also directly sensed by the hypothalamus and carry out a central role via hypothalamic mechanisms on insulin action and on stimulating hepatic glucose production (Yi et al. 2010; Qatanani and Lazar 2007; Lam et al. 2005). However, it remains to be clarified where and the extent to which level fatty acids elicit this effect (Yi et al. 2010).

### Therapeutic opportunities for affecting insulin sensitivity through central nervous system stimulation

Table 1 summarizes the many opportunities for glucose control and diabetes management arising from modulating the activity of the central nervous system, which could be used in the future as therapies through bioelectronic medicine. Different central pathways are involved in regulating the same processes in the liver and other peripheral tissues. In particular, this review has focused on the brain-related mechanisms and neuronal populations that control hepatic glucose production and peripheral glucose uptake as both of them have an impact on insulin sensitivity (*S*_*I*_).

**Table 1:** Central mechanisms involved in glucose homeostasis and benefit of modulating them for diabetes management

Region	Population of neurons	Peripheral mechanisms	Impact on glucose homeostasis	Ref.	Opportunities for diabetes management
ARC	AgRP	Food entrained oscillator	Regulate feeding behaviour	(Stephan et al. 1979)	Predicting cephalic phase
	*AgRP*→*aBNST*6^*vl*^	*↑* BAT myostatin expression	Insulin resistance	(Steculorum et al.^2016^)	Control of *S*_*I*_
	*ARC*^*AgRP*^→*LHN*	*↑* Appetite	*↑* *G*(*t*)	(Steculorum et al.^2016^)	Control of food intake
	AgRP^IR^	*↓* Hepatic vagal tone	*↓* HGP ⇔ Peripheral glucose uptake	(Shin et al. 2017; Yi et al. 2010; Scherer et al. 2011)	Control of hepatic *S*_*I*_
	NPY	*↑* Appetite	*↑* *G*(*t*)	(Billington and Levine 1992)	Control of food intake
		*↑* Hepatic sympathetic tone	*↑* HGP	(Yi et al. 2010)	Control of hepatic *S*_*I*_
	*NPY*→*PVN*^*TH*^	*↓* Sympathetic tone	*↓* BAT thermogenesis	(Yi et al. 2010; Shi et al. 2013)	-
	*POMC*	*↑* Sympathetic tone	*↑* WAT lypolisis	(Shin et al. 2017; Brito et al. 2007)	-
		MC4R mutations	Insulin resistance	(Elmquist and Marcus 2003)	Control of *S*_*I*_
	*P* *O* *M* *C* ^*IR*^	Impaired insulin action	*↑* WAT lipolysis	(Shin et al. 2017)	Control of *S*_*I*_
	*TH*	*↑* Appetite	*↑* *G*(*t*)	(Zhang and van den Pol 2016)	Control of food intake
VMH	*V* *M* *H* ^*SF*1^	-	*↑* Glucose uptake *↑* HGP	(Coutinho et al. 2017)	Control of hepatic *S*_*I*_
		*↑* Glucagon ⇔ Insulin	*↑* *G(t)*	(Shimazu and Ishikawa 1981; Stanley et al.^2016^; Shimazu and Ishikawa 1981)	Control of hypoglycemia
	*V* *M* *H* ^*Glut*^	*↑* Sympathetic tone	*↑* HGP *↑* Tissue uptake	(Shimazu et al. 1966; Gellman et al. 1981; Coutinho et al. 2017)	Control of blood glucose levels
		*↓* Glutamatergic metabolism	*↓* Response to hypoglycemia	(Chowdhury et al.^2017^)	Control of hypoglycemia
LHN	LHN	*↑* Parasympathetic tone	*↓* HGP	(Shimazu et al. 1991)	Control of blood glucose levels
	*L* *H* *N* ^*MC*4 *R*^	*↑* Sympathetic tone	*↑* BAT glucose uptake	(Morgan et al. 2015)	Control of *S*_*I*_
SCN	*SCN*→*PVN*	*↑* Sympathetic tone	*↑* HGP	(Fujii et al. 1989; Kalsbeek et al. 2004)	Control of hepatic *S*_*I*_

This table summarizes the impact of activating the most relevant brain regions involved in glucose homeostasis and the potential benefit of changing their activity for modulating insulin sensitivity (*S*_*I*_) and food intake in the context of type 1 and type 2 diabetes management. In particular, their effect on plasma glucose levels (G(t)), hepatic glucose production (HGP) and glucose uptake is shown

In diabetes, activation or inhibition of the neural activity of these neuronal populations (see Table 1) could be used to achieve glucose homeostasis. For example, through modulation of the neural signals we could control the course of insulin-independent and insulin-dependent mechanisms (i.e. influencing the effectiveness of insulin, *S*_*I*_) of hepatic glucose production and glucose uptake to reach optimal glucose control. This is very important for type 1 diabetes, where there is not an endogenous production of insulin from the pancreas. In these subjects, the insulin that is needed to lower glucose levels after a meal intake is externally delivered by either an insulin pen or a pump. In this case, modulation of the neural signalling pathways could increase the effect of exogenous insulin in lowering blood glucose and we would be able to reduce post-prandial glucose excursions, therefore reducing hyperglycemia. In addition, external stimulation of hypothalamic regions such as the VMH glutamatergic circuitry could help to reduce hypoglycemic events in type 1 diabetic subjects. This would be achieved as its activation promotes activation of hepatic gluconeogenesis and glucagon release from the pancreas, which elevate blood glucose levels.

As it has been previously explained and outlined in Table 1, the brain regions that have been presented in this review exert their action through sympathetic and parasympathetic pathways to the organs. Therefore, the same impact on glucose homoeostasis and diabetes control could be achieved by directly modifying their firing activity. Despite the potential benefits of using bioelectronic medicine in this sense, there are still many limitations that need to be overcome before becoming a reality in diabetes treatment. Among them, acquiring a full understanding of the exact relationship between the activity of the brain regions and their impact on glucose levels stands out.

## Review of the peripheral neural mechanisms that directly control the endocrine pancreatic function

As previously mentioned, glucose-sensing cells located at different anatomical sites, including the oral cavity, the gut, the hepatoportal vein area, the brainstem and hypothalamus are linked together through nervous connections to ultimately control the activity of the autonomic nervous innervation of the endocrine pancreas. This innervation not only controls glucagon and insulin secretion but also *α* and *β*-cell number and proliferation (Thorens 2011; 2010).

**Fig. 2:**
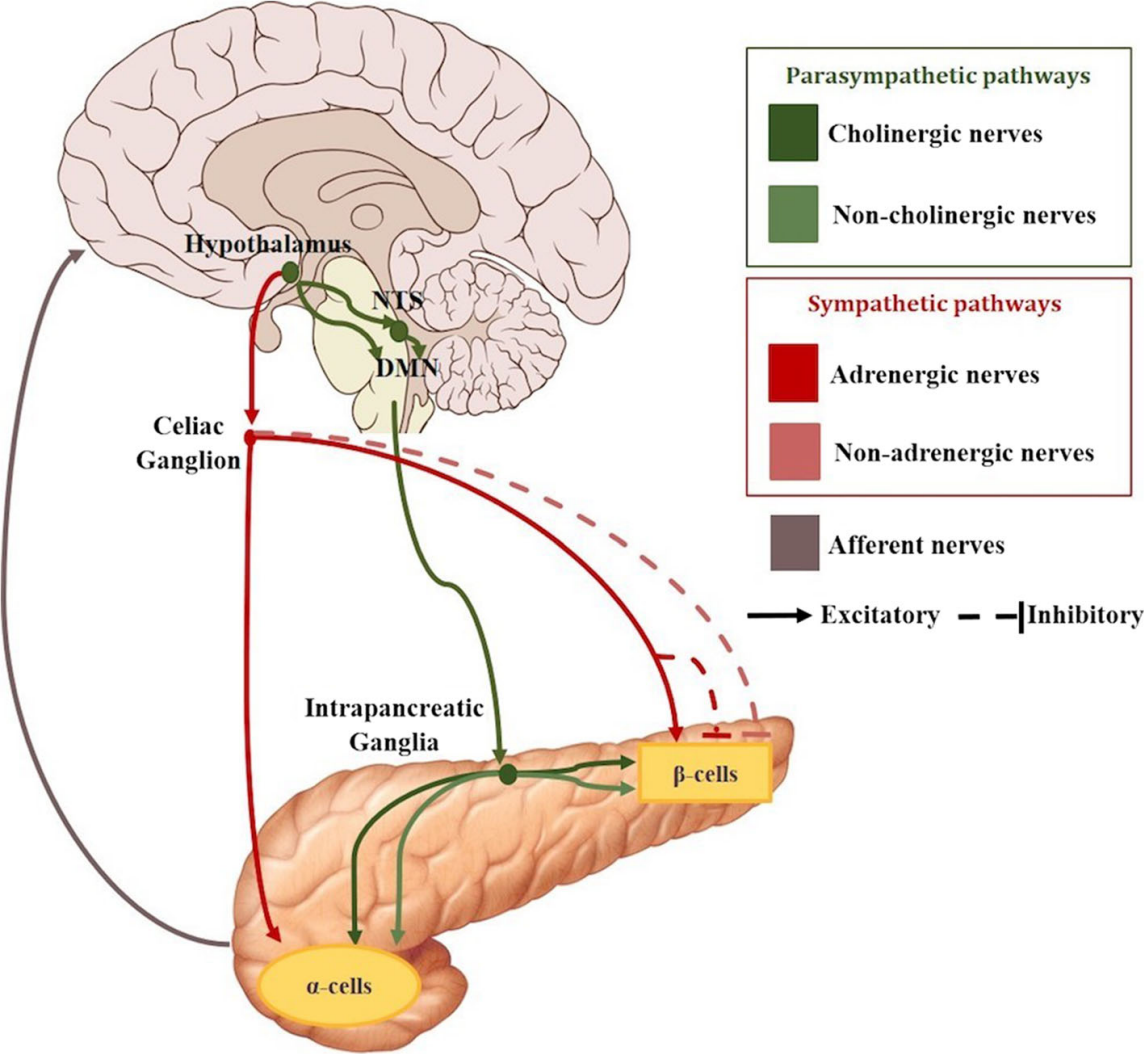
Schematic of the most significant neural connections between the brain and the pancreas. The neural pathways to the *α* and *β* cells include postganglionic parasympathetic (green color range) and sympathetic (red color range) nerves. Afferent connections from the pancreas to the brain are also depicted (grey)

There are two major pathways identified in regulating islet secretion, parasympathetic and sympathetic (see Fig. 2). There are other branches formed by sensory nerves, nitric oxide and CCK nerves that are also suggested to take part in regulating the pancreatic function, but the mechanisms are unclear (Ahrén 2000).

Two main parasympathetic mechanisms can be found: cholinergic regulation via release of acetylcholine (ACh) and non-cholinergic mechanisms mediated by neuropeptides such as vasoactive intestinal polypeptide (VIP), pituitary adenylate cyclase activating polypetide peptide (PACAP), or gastrin-releasing peptide (GRP). These neurotransmitters bind to specific receptors that are present on *β*-cells and *α*-cells.

Regarding the cholinergic pathway, it is formed by preganglionic fibres that originate in the dorsal motor nucleus of the vagus nerve (DMNX). These fibres then travel to the pancreas within the bulbar outflow tract and the hepatic and gastric nerves of the vagus. They synapse afterwards with cholinergic postganglionic fibres in the intrapancreatic ganglia. These nerves finally reach the islets enhancing insulin and glucagon secretions (Thorens 2011; Berthoud and Powley 1990; Gilon and Henquin 2001). Retrograde transsynaptic labeling with pseudorabies viruses has allowed to acquire a better understanding of the parasympathetic neuronal circuit innervating the pancreas and a link has been reported between the vagal nerve and the paraventricular nucleus (Thorens 2011; Jansen et al. 1997). These regions are also directly innervated by arcuate nucleus NPY and POMC neurons, suggesting a great implication of this area in the parasympathetic control of insulin secretion. In fact, intracerebroventricular administration of NPY rapidly induces insulin secretion through mechanisms that can be suppressed by vagotomy (Thorens 2011). When the vagus nerve is stimulated, the terminals of the preganglionic nerves in the intrapancreatic ganglia, release acetylcholine to the synapse within the postganglionic nerves. The latter have nicotinic receptors that bind with the acetylcholine, which in turn causes the release of acetylcholine from their terminals within the islets (Sha et al. 2001; Stagner and Samols 1986).

Focusing on insulin secretion, many studies have described that electrical stimulation of the vagus nerve, in conditions of slight hyperglycemia, stimulates its release from the *β*-cells (Berthoud et al. 1980; Kaneto et al. 1975; Frohman et al. 1967). This has to do with the fact that pancreatic *β*-cells present muscarinic receptors (m3AChR) that bind to the released acetylcholine eliciting the hormone secretion (Ahrén 2000; Berthoud and Powley 1990; Gautam et al. 2006; Verspohl et al. 1990). The parasympathetic tone to the endocrine pancreas can also be increased by lesions of the ventromedial hypothalamus, which lead to increased *β*-cell proliferation and mass (Niijima 1986; Kiba 2004; Kiba et al. 1996), confirming that both total *β*-cell number and secretion activity can be regulated by vagal control.

**Fig. 3:**
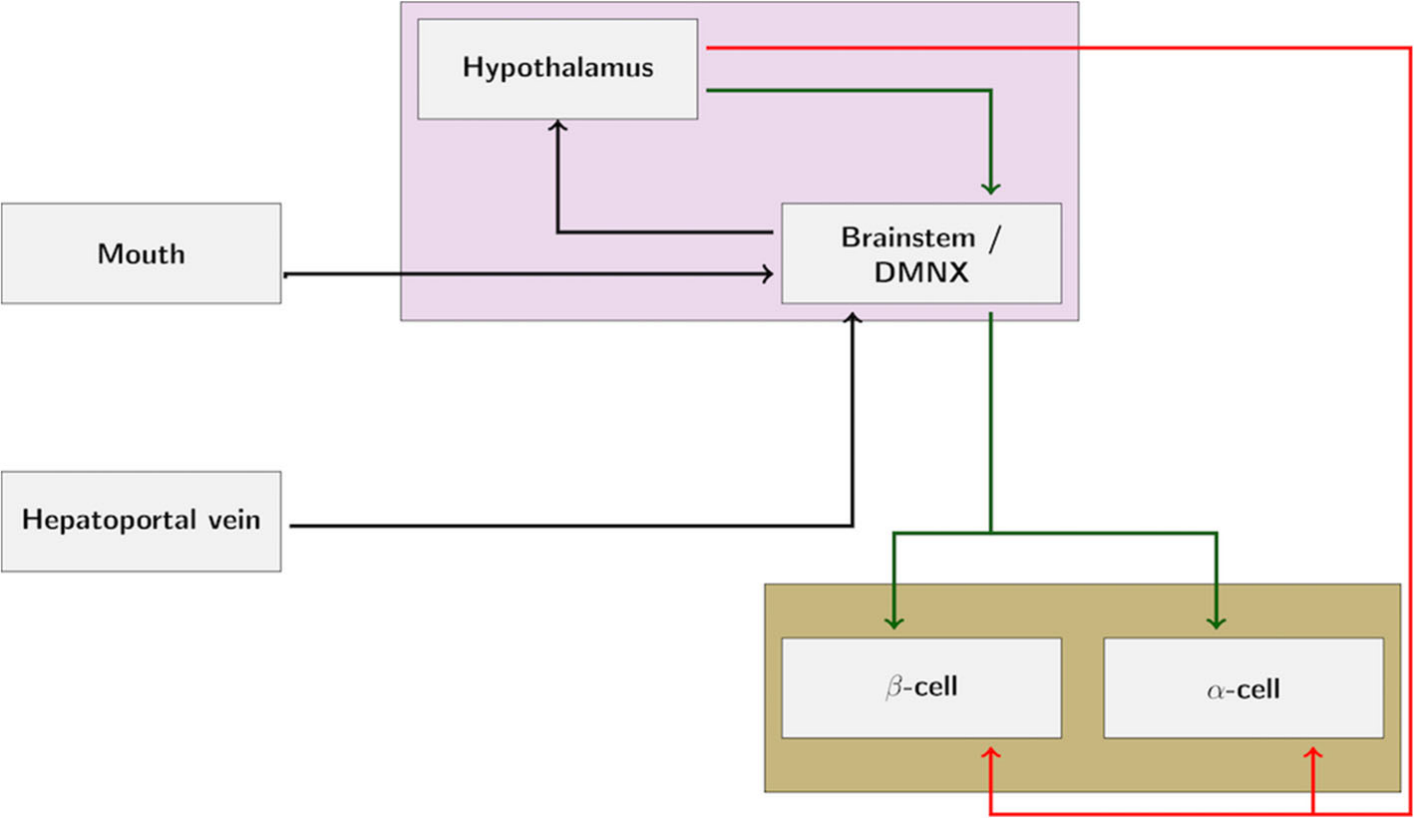
Schematic of the nervous reflex involving neural glucose sensing that controls pancreatic secretion. Glucose in the oral cavity and in the hepatoportal vein area induces pancreatic secretion by mechanisms that integrate afferent nerves that project from the taste buds and the hepatoportal vein to the brainstem and hypothalamus and efferent nerves from these areas to the pancreas. Green lines: parasympathetic innervation (via release of the neurotransmitters ACh, VIP, PACAP, GRP); Red lines: sympathetic innervation (via release of the neurotransmitters NE, NPY, galanin)

In particular during the first phase of secretion, a nervous reflex involving glucose sensing from different locations is key in enhancing insulin release (Thorens 2011; Berthoud et al. 1981). As an example, glucose in the oral cavity and, after its intestinal absorption, in the hepatoportal vein area, induces insulin secretion by a mechanism that increases the firing rate of vagal afferent nerves that project from the taste buds and the hepatoportal vein respectively to the brainstem and hypothalamus, which can be suppressed by vagotomy (see Fig. 3) (Berthoud et al. 1981; Berthoud et al. 1980; Berthoud and Jeanrenaud 1982; Thorens 2001). Moreover, as previously described, glucose injections in neurons of the arcuate and paraventricular nuclei have also been found to activate insulin secretion (Guillod-Maximin et al. 2004). Likewise, there are studies that reveal an increase in the secretion of glucagon after vagal stimulation in several species (Niijima 1986; Verspohl et al. 1990) including humans (Thorens 2011; Bloom et al. 1974). Parasympathetic non-cholinergic mechanisms are also suggested to contribute to the release of both insulin and glucagon in vivo and in vitro in several species including humans (Ahrén 2000). Thus, it appears that both cholinergic and non-cholinergic mechanisms enhance islets’ hormone secretions through vagal stimulation (Ahrén 2000).

Focusing now on the sympathetic pathways, sympathetic nerve endings contain norepinephrine (NE), called adrenergic nerves, but also neuroptide Y and galanin, called non-adrenergic nerves. Sympathetic preganglionic nerve fibres originate in the hypothalamus and leave the spinal cord at levels C8 to L3. Then, they pass through the lesser and greater splanchnic nerves to reach the celiac and mesenteric ganglia, where they synapse with the postganglionic fibres which enter the pancreas along the blood vessels. However, preganglionic sympathetic nerve fibres can also directly enter the pancreas without a ganglionic synapse (Ahrén 2000; Gilon and Henquin 2001; Brunicardi et al. 1995). There are two major sympathetic pathways: the adrenergic and non-adrenergic pathways.

Electrical stimulation of adrenergic nerves have been demostrated to elicit a large release of noradrenaline into the pancreatic veins, which act through *α*-adrenoceptors to inhibit both basal and glucose-stimulated insulin secretion (Thorens 2011; Verspohl et al. 1990). On the other hand, noradrenaline can also stimulate insulin secretion by two different actions. Firstly, through the activation of the islet *β*2-adrenoceptors, which stimulates insulin secretion. Secondly, through direct action on the *α*-cells which stimulates glucagon secretion, that, in turn, also eventually stimulates insulin release. Therefore, the net effect of noradrenaline on insulin secretion might depend on the relative activity of the two types of receptors on the *β*-cells and on the action through glucagon (Westfall 1984), but the overall effect is an inhibition of insulin release. The non-adrenergic influence of the inhibitory action of sympathetic nerves on insulin secretion is thought to be mediated by galanin and NPY. However, after an electrical activation of the sympathetic nerves, these neuropeptides have a great range of responses depending on the species (Thorens 2011; Ahrén 2000).

The sympathetic nervous system plays a predominant role in stimulating glucagon secretion, through activation of the *α*-cell *β*2-adrenergic receptors (Thorens 2011; Verspohl et al. 1990). Similarly to the nervous reflex described in insulin secretion, there are studies suggesting a direct involvement of both central and peripheral glucose sensors in the control by the autonomic nervous system of *α*-cell secretion activity (see Fig. 3) (Thorens 2011). In particular, activation of the sympathetic response depends on hypoglycemia detection at several sites. For example, the hepato-portal vein region contains glucose sensitive nerve terminals that can control glucagon secretion as indicated by the fact that portal vein glucose infusion can suppress hypoglycemia-induced increase in plasma glucagon (Hevener et al. 2000).

### Therapeutic opportunities for affecting pancreatic endocrine function through peripheral nerve stimulation

Table 2 summarizes the opportunities that exist for improving glucose control by modulating the neural information that the brain sends to the pancreas. This can be particularly desirable in situations where glucose control in diabetic subjects is challenging with exogenous insulin or oral medications. Examples of these situations include ingestion of a meal which would cause glucose to rise or exercise which would cause glucose to lower. In the latter, for example, a prompt inhibition of insulin secretion and a promotion of secretion of counterregulatory hormones (such as glucagon or epineprhine) before starting exercise can considerably improve the control of glucose levels and reducing the risk of hypoglycemic events, especially for type 2 diabetes. In addition, hypoglycemia is usually a side effect of treatment with blood-sugar-lowering medication, such as insulin therapies in both types of the disease (Zammitt and Frier 2005; McCrimmon and Sherwin 2010). This limitation can be overcome by initiating a fast response that increases glucose levels. Moreover, in type 1 diabetes patients, meal intake surprisingly results in an increase of glucagon secretion, which contributes to hyperglycemia and makes the control of glucose fluctuations challenging (Hughes and Narendran 2014). Some studies have hypothesized that this occurs because there is not an increase of insulin secretion that makes the *α*-cells reduce the release of glucagon (Abdul-Ghani and DeFronzo 2007). Therefore, inhibition of the neural pathways that promote glucagon secretion or stimulating those that inhibit its release when food is ingested, could allow improved control of glucose during meals in these patients.

**Table 2:** Peripheral pathways involved in pancreatic secretion and benefit of modulating them for diabetes management

Peripheral pathway	NT	Pancreatic receptor	Impact on endocrine pancreas	Impact on glucose homeostasis	Opportunities for diabetes management
Parasympathetic	ACh	Muscarinic receptors (m3AChR)	*↑* insulin *↑* glucagon *↑* *β*-cell number	*↓* postprandial glucose levels	Tighter control of glucose levels and preparation of the body to meals
	VIP, PACAP, GRP	VIP, PACAP, GRP	*↑* hormonal secretion (less studied)	-	-
Sympathetic		*β*-cells *α*-adrenoreceptors	Inhibit basal & glucose-dependent insulin secretion	*↑* glucose levels	Reduction of hypoglycemia
	NE	*β*-cells *β*-adrenoreceptors	*↑* insulin secretion	*↓* glucose levels	Reduction of hyperglycemia
		*α*-cells *β*2-adrenoreceptors	*↑* glucagon *↑* insulin	*↓* glucose levels	Control of glucose homeostasis
	Galanin & NPY	Specific receptors	Great range of responses	-	-

This table shows the peripheral pathways that are most involved in the pancreatic secretion and the neurotransmitter (NT) involved in each of them. The effect of increasing their basal tone on glucose homeostasis and the potential benefits of modulating their firing pattern for diabetes management are summarised as well

In this context, further research to specifically activate the type of receptors that cause the desire response is needed. This is particularly important in stimulating or inhibiting the sympathetic nerves, because the observed outcome in glucose greatly varies depending on the receptor to which noradrenaline is linked (see Table 2).

## Conclusion

In this review, a general overview of all the neural mechanisms by which the brain directly and indirectly controls glucose homoeostasis have been presented as a new perspective for studying metabolic diseases. In particular, the nervous system has a significant role in i) the liver, by managing glucose production, lipolysis and glucose uptake, ii) the glucose utilization by peripheral tissues, including the brown and white adipose tissue and muscle, and iii) the hormone secretion by the endocrine pancreas. In addition, the possibility of modulating insulin sensitivity through chemical and/or electrical stimulation of particular brain regions (particularly in the hypothalamus) and autonomic nerves (both sympathetic and parasympathetic) has been given particular consideration.

To date, there is only a rough understanding of the physiological mechanisms beneath this neural-metabolic interaction. Hence, more research is needed to develop the previously mentioned therapies, starting from establishing the direct quantitative effect of neural stimulation on both the insulin sensitivity and pancreatic secretion (Tang et al. 2018). Such studies are also expected to increase our knowledge of the neural mechanism of insulin sensitivity and pancreatic secretion, and consequently, glucose homoeostasis.

This review provides the starting point for the development of new therapies in the field of bioelectronic medicine for diabetes management. We foresee that in the future miniaturized devices will be implanted at selective nerve fibres or brain areas to modulate their electric signals and treat this endocrine disorder. In fact, over the last years novel treatment strategies for type 2 diabetes based on electrical stimulation of peripheral nerve fibres have been investigated. The work in this field has mainly occurred in terms of establishing the effect of the neurostimulation in food intake (Zhang and van den Pol 2016; Steculorum et al. 2016) and, recently, in restoring insulin sensitivity (Sacramento et al. 2018). We propose to extend this idea by using well defined electrical signals to modulate insulin sensitivity for type 1 diabetes treatment. In addition, the neural information obtained from recordings either from specific brain regions or peripheral nerves can be incorporated as an input in current close-loop controllers (Herrero et al. 2012). This may significantly improve glucose control, in particular during difficult-to-control events such as meals. We hope that our focused effort in this new emerging field will stimulate new ideas and directions in diabetes management.

## Abbreviations

*α*-MSH: *α*-melanocite-stimulating hormone
ACh: Acetylcholine
ACTH: Adrenocorticotropic hormone
AP: Artificial pancreas
APF: Action potential frequency
ARC: Arcuate nucleus of the hypothalamus
BAT: Brown adipose tissue
BCAA: Branched-chain amino acids
BCKDH: Branched-chain alpha-keto acid dehydrogenase
CNS: Central nervous system
DMNX: Dorsal motor nucleus of the vagus nerve
FA: Fatty acid
G(t): Plasma glucose levels
GE: Glucose excited
GI: Glucose inhibited
GIR: Glucose infusion rate
GRP: Gastrin-releasing polypeptide
HGP: Hepatic glucose production
HPT: Hypothalamic-pituitary-thyroid
LC: Locus coeruleus
LHN: Lateral hypothalamic nucleus
MC2R: Melanocortin 2 receptor
MC3R: Melanocortin 3 receptor
MC4R: Melanocortin 4 receptor
Mstn: Myostatin
NE: Norepinephrine
NEFAs: Non-essential fatty acids
NPY/AgRP: Neuropeptide Y/agouti-related peptide
NTS: Nucleus of the tractus solitarius
PACAP: Pituitary adenylate cyclase activating polypeptide
PEPCK: Phosphoenolpyruvate carboxykinase
PK: Piruvate kinase
POMC/CART: Pro-opiomelanocortin/cocaine-amphetamine related transcript
PVN: Paraventricular nucleus
SCN: Suprachiasmatic nucleus
SI: Insulin sensitivity
SNA: Sympathetic nerve activity
TH: Tirosine hydroxylase
VIP: Vasoactive intestinal polypeptide
VMH: Ventromedial nucleus of the hypothalamus
WAT: White adipose tissue
